# A novel disulfidptosis-related mRNA signature predicts prognosis and therapeutic response in lung squamous cell carcinoma

**DOI:** 10.1186/s12890-025-03920-6

**Published:** 2025-10-08

**Authors:** Wei Bai, Ning Jiang, Yuhan Deng, Xiaofeng Tang, Feifei Zhang, Shaorui Niu, Yuyang Yao, Yuhao Zhou, Kangming Chen, Liping Li, Jun Yang, Xiao-Bin Lv

**Affiliations:** 1 School of Public Health, Jiangxi Medical College, Nanchang University, Nanchang330006, China; 2 Jiangxi Provincial Key Laboratory of Disease Prevention and Public Health, Nanchang University, Nanchang330006, China; 3 Jiangxi Key Laboratory of Oncology, The Third Affiliated Hospital, Jiangxi Medical College, The Central Lab of The First Hospital of Nanchang, Nanchang University, North 128 Xiangshan Road, Nanchang, 330008 China; 4 Institute (College) of Integrative Medicine, Dalian Medical University, Dalian, 116044 China; 5 College of Integrated Chinese and Western Medicine, Hunan University of Chinese Medicine, Changsha, 410208 China

**Keywords:** Lung squamous cell carcinoma, Disulfidptosis, Prognostic signature, Risk stratification, Tumor microenvironment

## Abstract

**Background:**

Lung squamous cell carcinoma (LUSC) remains an aggressive malignancy with limited therapeutic options and poor prognosis. Recent studies have identified disulfidptosis as a novel form of metabolic stress-induced cell death, but its clinical implications in LUSC remain unexplored. This study investigates the prognostic value of disulfidptosis-related genes (DRGs) in LUSC.

**Methods:**

We analyzed transcriptomic data from TCGA-LUSC cohort and identified DRGs through intersection with established disulfidptosis-related gene sets. A protein-protein interaction (PPI) network was constructed, and univariate Cox regression was performed to select prognostic genes. A risk score model was developed using multivariate Cox regression. The model’s performance was evaluated using ROC curve and Kaplan-Meier analyses. Functional enrichment and immune microenvironment analyses were conducted to explore potential mechanisms.

**Results:**

We identified 9 prognostic DRGs (FHOD1, ORC5, TRIR, ALKBH1, EPS8L2, MBLAC1, MYADM, HTRA2, and SRI) that significantly correlated with patient survival. The risk score model effectively stratified patients into high- and low-risk groups (*P* < 0.001), with C-index values of 0.78 at 1 year and 0.75 at 3 years. High-risk patients showed enriched cytokine-cytokine receptor interactions and immunosuppressive microenvironments, while low-risk patients exhibited activated metabolic pathways. Experimental validation confirmed ORC5’s oncogenic role in promoting proliferation and invasion.

**Conclusion:**

We established a novel 9-gene prognostic signature based on disulfidptosis-related genes that effectively predicts LUSC outcomes. These findings highlight the clinical relevance of disulfidptosis in LUSC and provide potential biomarkers for risk stratification and therapeutic targeting.

**Supplementary Information:**

The online version contains supplementary material available at 10.1186/s12890-025-03920-6.

## Introduction

Lung cancer remains the leading cause of cancer-related mortality worldwide, with non-small cell lung cancer (NSCLC) representing approximately 85% of cases [1]. Among NSCLC subtypes, lung squamous cell carcinoma (LUSC) accounts for 20%−30% of diagnoses and is characterized by high genomic instability, limited targeted therapy options, and a dismal 5-year survival rate below 15% [2, 3]. These clinical challenges underscore the urgent need for reliable prognostic biomarkers and novel therapeutic targets to improve the management of LUSC.

Regulated cell death (RCD) pathways, including ferroptosis and pyroptosis, have emerged as critical regulators of tumor progression and therapy resistance [4–6]. Notably, targeting RCD-related genes (e.g., inhibiting ferroptosis to suppress LUSC growth) has shown promising therapeutic potential [7]. Recently, a novel RCD subtype termed disulfidptosis was identified, wherein glucose starvation triggers aberrant disulfide bonding in actin cytoskeletal proteins, leading to catastrophic cell death [8]. Growing evidence implicates disulfidptosis in cancer progression, with disulfidptosis-related gene (DRG) signatures demonstrating prognostic value in pancreatic cancer and clear cell renal cell carcinoma [9, 10].

Despite these advances, the clinical relevance of disulfidptosis in LUSC remains to be explored. In this study, we constructed a DRG-derived mRNA signature using transcriptomic data from the TCGA-LUSC cohort. Our study not only predicts patient survival but also correlates the signature with tumor immune microenvironment features and chemotherapy response, offering an actionable tool for personalized LUSC therapy.

## Materials and methods

### Data acquisition

We obtained patient data from the TCGA database (https://portal.gdc.cancer.gov), which included a total of 553 samples, comprising 51 normal samples and 502 tumor samples. Additionally, we collected clinical data from 504 samples, which were used for subsequent prognostic analysis. Furthermore, we gathered mutation data related to lung squamous cell carcinoma (LUSC) from the TCGA database to facilitate comprehensive molecular and clinical investigations.

### Establishing a prognostic signature for disulfidptosis-related mRNAs

We identified 24 disulfidptosis-related genes (DRGs) (ACTB, ACTN4, CAPZB, CD2AP, DSTN, FLNB, FLNA, GYS1, INF2, IQGAP1, LRPPRC, MYH10, MYH9, MYL6, NCKAP1, NDUFA11, NDUFS1, NUBPL, OXSM, PDLIM1, RPN1, SLC3A2, SLC7A11, and TLN1) from relevant studies [11, 12]. Using the R package “limma,” we screened for mRNAs associated with disulfidptosis by applying the criteria of |Cor| >0.4 and *P* < 0.001. We subsequently employed the R packages “dplyr,” “ggalluvial,” and “ggplot2” to construct Sankey diagrams, which visually illustrate the coexpression relationships between the identified mRNAs and DRGs (Supplementary Fig. S1).

### Determination of TRAIN and TEST cohorts

After excluding samples with unknown survival times, we retained 495 clinical samples, which were randomly divided into two groups: the training cohort (*n* = 248) and the testing cohort (*n* = 247). Chi-square tests were then performed to compare the clinical characteristics across all, test, and training cohorts, and the results are shown in Table 1. The p-values for all these clinical features were > 0.05, indicating no significant differences in the baseline characteristics between the three groups. This demonstrates the random distribution of these features across groups and validates the appropriateness of our grouping strategy.

**Table 1:** Comparison of clinical characteristics

Covariates	Type	Total	Test	Train	*P*-value
Age	<=65	189(38.18%)	86(34.82%)	103(41.53%)	0.1681
Age	> 65	300(60.61%)	157(63.56%)	143(57.66%)	
Age	unknow	6(1.21%)	4(1.62%)	2(0.81%)	
Gender	FEMALE	129(26.06%)	68(27.53%)	61(24.6%)	0.5215
Gender	MALE	366(73.94%)	179(72.47%)	187(75.4%)	
Stage	Stage I	242(48.89%)	119(48.18%)	123(49.6%)	0.8851
Stage	Stage II	159(32.12%)	82(33.2%)	77(31.05%)	
Stage	Stage III	83(16.77%)	39(15.79%)	44(17.74%)	
Stage	Stage IV	7(1.41%)	4(1.62%)	3(1.21%)	
Stage	unknow	4(0.81%)	3(1.21%)	1(0.4%)	
T	T1	114(23.03%)	63(25.51%)	51(20.56%)	0.3529
T	T2	288(58.18%)	144(58.3%)	144(58.06%)	
T	T3	70(14.14%)	31(12.55%)	39(15.73%)	
T	T4	23(4.65%)	9(3.64%)	14(5.65%)	
M	M0	407(82.22%)	211(85.43%)	196(79.03%)	1
M	M1	7(1.41%)	4(1.62%)	3(1.21%)	
M	unknow	81(16.36%)	32(12.96%)	49(19.76%)	
N	N0	316(63.84%)	153(61.94%)	163(65.73%)	0.3999
N	N1	128(25.86%)	69(27.94%)	59(23.79%)	
N	N2	40(8.08%)	19(7.69%)	21(8.47%)	
N	N3	5(1.01%)	1(0.4%)	4(1.61%)	
N	unknow	6(1.21%)	5(2.02%)	1(0.4%)	

### Predictive model construction and validation

First, we performed univariate Cox analysis to screen all the mRNAs associated with LUSC prognosis. The “glmnet” package in R was used to select predictive mRNAs via the Least Absolute Shrinkage and Selection Operator (LASSO) method, with the optimal regularization parameters determined through 10-fold cross-validation [13]. Finally, multivariate Cox regression analysis was conducted to identify the mRNAs most strongly associated with LUSC.

A risk score formula was then established on the basis of the selected mRNAs [risk score = (coef mRNA1 × expr mRNA1) + (coef mRNA2 × expr mRNA2) +… + (coef mRNAn × expr mRNAn)], where “coef” refers to the regression coefficient from the multivariate Cox analysis, and “expr” represents the expression level of the corresponding mRNA in the samples. The risk scores for all samples were calculated, and on the basis of the median value, the samples were divided into a low-risk group (LRG) and a high-risk group (HRG). To validate the accuracy of our model, survival analysis was performed for different cohorts (ALL, TRAIN, and TEST), including progression-free survival (PFS), mRNA-related heatmaps, and survival scatter plots.

### Prognostic analysis of the model

We conducted both univariate and multivariate Cox regression analyses on the clinical data to assess whether the risk score could independently serve as a prognostic factor. The accuracy of the risk score model was then evaluated via ROC curve analysis and C-index analysis.

Finally, we used Kaplan‒Meier (K‒M) curves to assess the applicability of the model. For example, we grouped patients on the basis of stage (I‒IV), sex (male vs. female), age (> 65 vs. ≤65), and N stage (N0 vs. N1‒N3, where N0 indicates no tumor spread and N1‒N3 indicates tumor progression). By observing the prognostic differences between the high-risk group (HRG) and low-risk group (LRG) in these subgroups, we further validated the applicability of the model.

### Functional enrichment analysis

In order to explore the reasons behind the difference in prognosis between the two risk groups, we conducted a Functional enrichment analysis of the two risk groups. We used the “limma” R package to identify differentially expressed genes between the high-risk and low-risk groups, with the criteria of a |log2-fold change| >1 and a false discovery rate (FDR) < 0.05. We subsequently performed Gene Ontology (GO) enrichment analysis and Kyoto Encyclopedia of Genes and Genomes (KEGG) functional enrichment analysis on the DEGs via the “clusterProfiler” package, with *P* < 0.05 as the filtering threshold.

Finally, we conducted Gene Set Enrichment Analysis (GSEA) using the KEGG pathway gene set “c2.cp.kegg.Hs.symbols” (https://www.gsea-msigdb.org) to explore the differences in enriched pathways between the high-risk and low-risk groups.

### Immune microenvironment analysis

We used the R package “estimate” to score the tumor microenvironment from three aspects: stromal, immune, and ESTIMATE scores.

The immune infiltration status of the samples was assessed via the CIBERSORT algorithm. Additionally, we employed the “ssGSEA” algorithm from the “GSEA” R package to evaluate the immune functions of all the samples. The resulting data were merged with the risk group information, and the results were visualized as boxplots via the “ggpubr” R package.

### Analysis of TMB (tumor mutation burden)

We used the R package “maftools” to associate the risk groups with tumor mutation data, displaying the mutation frequency of genes across different risk groups. To clearly illustrate the direct differences in TMB between the risk groups, we visualized the data via violin plots.

Additionally, we used the R packages “survival” and “survminer” to study the impact of the risk score and TMB on the survival rates of the samples.

### Drug sensitivity analysis

We obtained drug sensitivity data from the GDSC database and used the R package “oncoPredict” to predict drug sensitivity differences between the high-risk group (HRG) and low-risk group (LRG). The screening criterion was set to *P* < 0.001.

### The expression pattern of ORC5

The primary reasons for using ORC5 as our in vitro experimental subject are as follows. Firstly, ORC5 has the most significant impact on poor prognosis in our predictive model. Secondly, existing studies have shown that ORC mRNA is significantly increased in lung adenocarcinoma tissues, and it is also an unfavorable prognostic factor in other cancers such as liver cancer and HPV-negative head and neck squamous cell carcinoma [14–16]. Lastly, ORC5 is closely related to DNA replication, which can play a regulatory role in the progression of cancer [17].We conducted differential expression analysis on samples from the TCGA database using TIMER2.0 and obtained plots showing the differential expression levels of ORC5 in lung tumor tissue versus normal tissue, as well as in other tumor tissues versus normal tissues.

### Cell transfection

Under conditions of 37 °C and 5% CO2, the normal human lung epithelial cell line BEAS-2B and the human lung squamous cell carcinoma (LUSC) cell line SK-MES-1 were cultured. The cells used in this study were obtained from the laboratory’s cell bank, where they were originally acquired from the Chinese Academy of Sciences Cell Bank. All cell lines were cultured according to standard protocols and regularly tested for mycoplasma contamination. Small interfering RNA (siRNA) targeting human ORC5 was obtained from Sigma. When the SK-MES-1 cells reached 50% confluence, the siRNA was mixed with Opti-MEM and Invitrogen’s transfection reagent and incubated for 20 min. The siRNA complex was then added to fresh medium. For plasmid transfection, the required amount of plasmid DNA was diluted in Opti-MEM, and Lipofectamine 2000 was mixed with an appropriate volume of Opti-MEM and incubated for 5 min. After mixing the DNA and transfection reagent, the mixture was left to stand for 10–20 min to form complexes, which were then immediately added to the culture dish. Six hours after transfection, the medium was replaced. After 24–48 h of transfection, the cells were harvested for validation.

### Quantitative real-time fluorescence PCR

The cells were seeded evenly in a 6-well plate. Once the cells reached 80% confluence, the medium was removed, and RNA was extracted via the TRIzol reagent from Takara. A cDNA synthesis mixture was prepared with 1 µg of template RNA, 2 µL of 8 × gDNA Eraser Premix, and 4 µL of 5 × RT Premix, resulting in a total volume of 20 µL with nuclease-free water. The reaction mixture was incubated at 37 °C for 10 min and then at 85 °C for 5 s to generate cDNA. RT‒qPCR amplification was performed using cDNA as the template. The reaction mixture (10 µL) was prepared in a 96-well plate containing 5 µL of 2× TB Green Premix Ex Tag II Fast qPCR reagent, 0.5 µL of forward and reverse primers, 1 µL of cDNA, and 3 µL of nuclease-free water. Three technical replicates were set for each sample. The cycling conditions were as follows: initial denaturation at 95 °C for 30 s, followed by denaturation at 95 °C for 10 s, annealing at 60 °C for 30 s, and extension at 95 °C for 15 s, followed by 60 °C for 60 s and 95 °C for 15 s. Primers for ORC5: 5′-TGAACCCGTGGTTAAAGGAG-3′ (forward) and 5′-CCCGGATCTGTGTCATCTTT-3′ (reverse); GAPDH primers: 5′-TGACTCATACAGCGACCCA-3′ (forward) and 5′-CACCCTGTGTGCTAGCAAA-3′ (reverse). Relative gene expression was calculated via the 2−∆Ct method, with GAPDH serving as the internal reference.

### Cell proliferation

Cell lines with ORC5 gene knockdown or overexpression, as well as their corresponding control cell lines, were seeded at 300 cells per well in a 96-well plate and cultured at 37 °C. After 24 h, 10 µL of Cell Counting Kit-8 (CCK-8) solution was added to each well and incubated for an additional hour. The cell viability was then measured via a microplate reader at 450 nm.

### Wound healing assay

Cell migration ability was assessed via a wound healing assay. The cells were scraped with the tip of a 200 µL pipette, washed three times with phosphate-buffered saline (PBS), and then incubated with serum-free medium at 37 °C. Two time points were set in the experiment: 0 h and 24 h. At both time points, images of the wound area were captured via an inverted microscope to observe cell migration and closure.

### Transwell assay

The cell invasion potential was measured via the following method. First, a mixture of Matrigel and serum-free medium (1:8) was added to the upper chamber of a 24-well plate and incubated in a sterile environment until the mixture solidified. Then, 4 × 10^4 cells were seeded into the upper chamber, which was cultured with 200 µL of serum-free medium, while 500 µL of medium containing 10% fetal bovine serum was added to the lower chamber. After 24 h, un-filtered cells in the upper chamber were gently removed via a cotton swab. The chamber was then immersed in 4% methanol and 0.1% crystal violet solution. Finally, images were captured under an inverted microscope.

### Colony formation assay

The cells to be tested were seeded into culture dishes and cultured until an appropriate density was reached. The cells were then diluted such that each well of a 6-well plate contained 1,000 cells. The cells were cultured for 7–14 days, with periodic observation to ensure that the colonies were large enough to be clearly visible and countable. Afterward, crystal violet staining was performed, and the number of colonies in each experimental group was counted.

### Statistical analysis

The bioinformatics analysis was performed via R software version 4.2.2. For the statistical analysis of the results of the molecular biology experiments, GraphPad Prism 10 was used. An unpaired t test was conducted to analyze the differences between two groups. For three or more groups, one-way ANOVA (both parametric and nonparametric) and the Kruskal‒Wallis test were applied as appropriate. A p value < 0.05 was considered statistically significant.

## Results

### Identification of disulfidptosis-related mRNAs

**Fig. 1:**
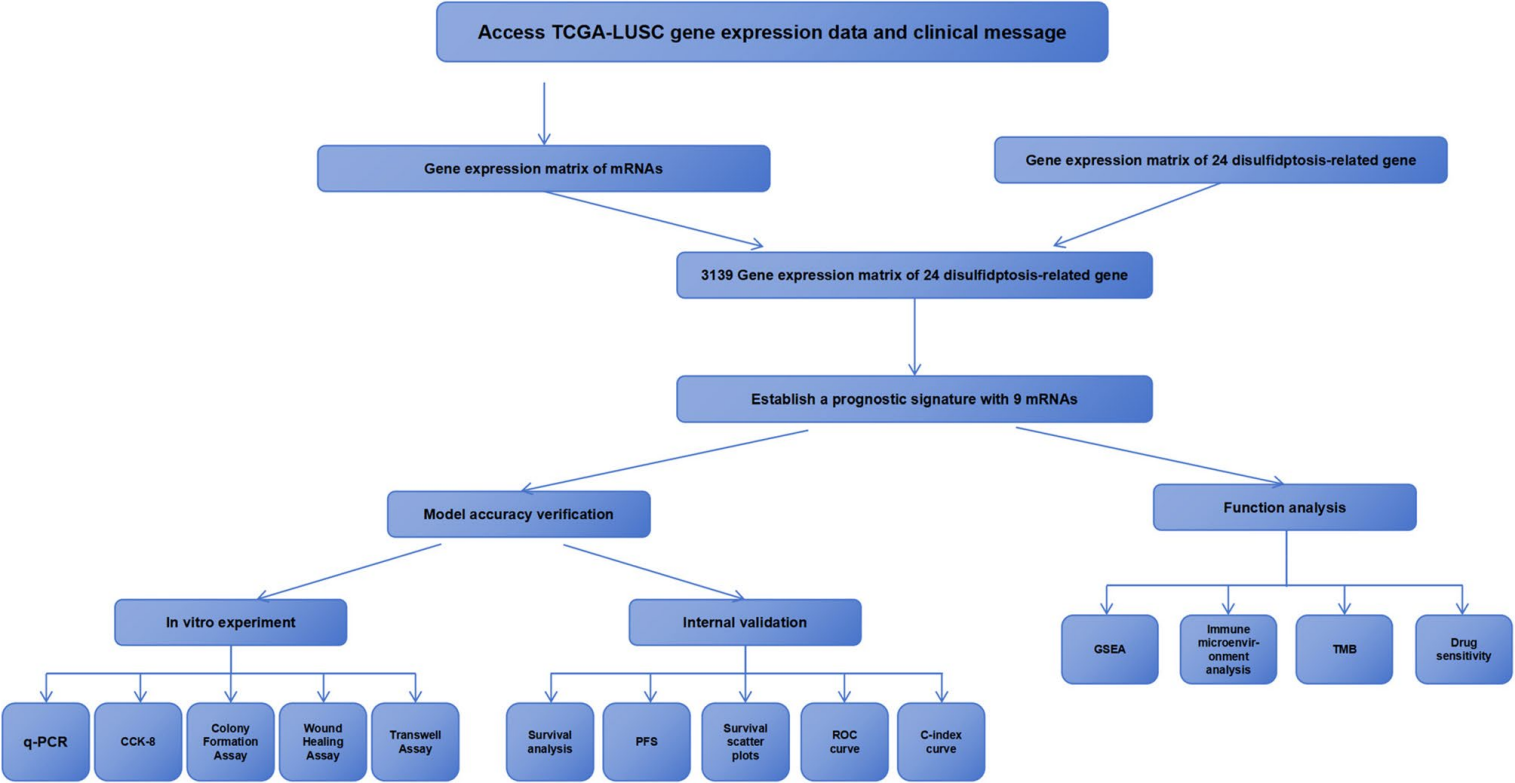
Study flow chart of the article

We extracted 3,139 disulfidptosis-related mRNAs (Supplementary Table S1) from 553 samples in the TCGA database on the basis of disulfidptosis genes identified in the published literature. The relationship between DRGs and the obtained mRNA was visualized. (Supplementary Fig. S1). Figure 1 illustrates the overall workflow of this study.

### Construction of the prognostic signature

**Fig. 2:**
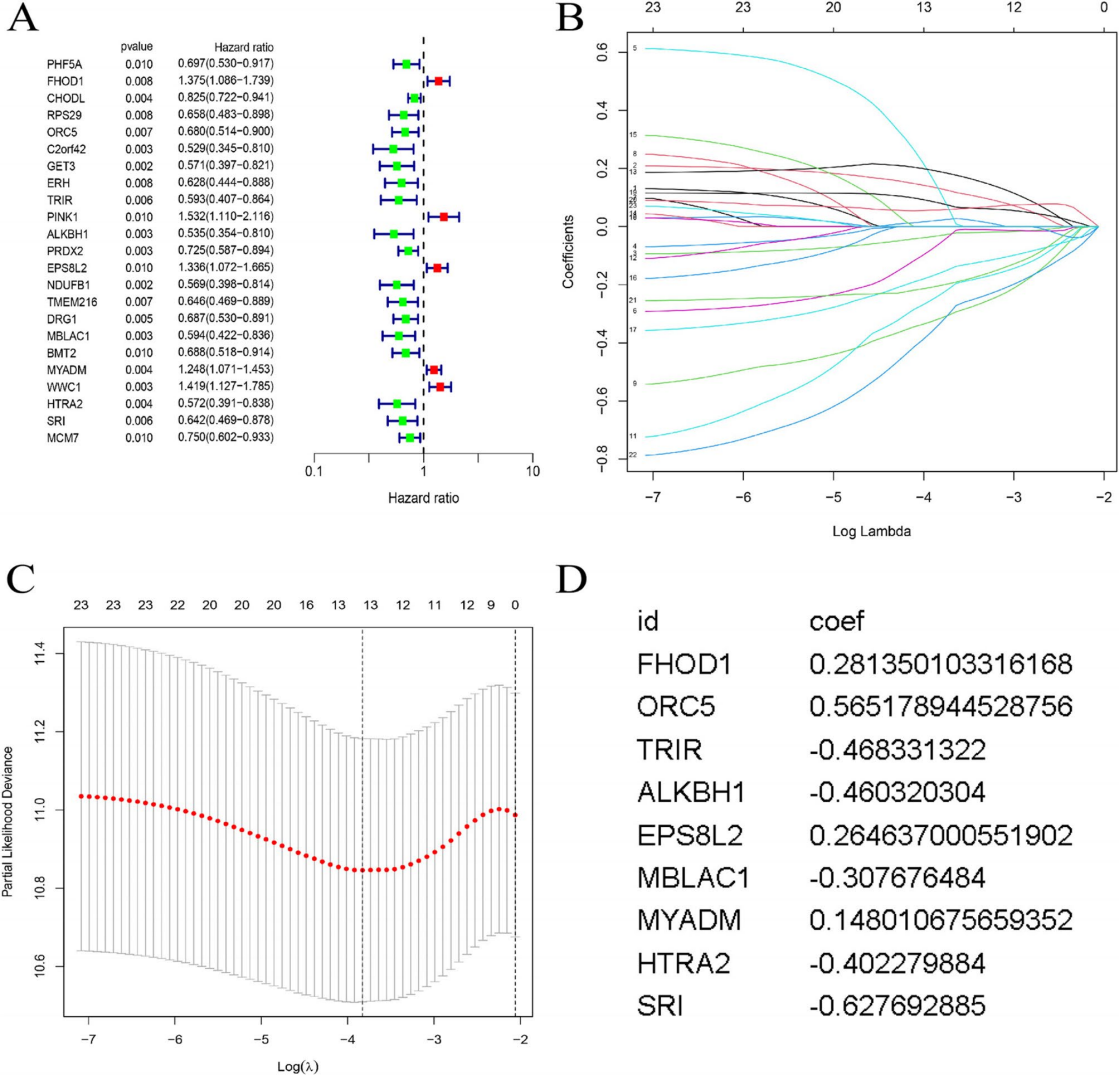
Construction of the prognostic signature. (**A**) Forest plot of the univariate Cox analysis results, where red indicates poor prognostic factors and green represents good prognostic factors. (B-C) Results of LASSO regression and tenfold cross-validation. (D) Coefficients from multivariate Cox regression analysis of key mRNAs

We performed univariate Cox regression analysis on disulfidptosis-related mRNAs and identified 23 genes associated with prognosis, which were visualized in a forest plot (Fig. 2A). Next, LASSO analysis was used to select 13 more reliable and predictive mRNAs (Fig. 2B, C). Finally, multivariate Cox regression analysis identified the 9 most prognostic mRNAs (FHOD1, ORC5, TRIR, ALKBH1, EPS8L2, MBLAC1, MYADM, HTRA2, and SRI) (Fig. 2D). These 9 mRNAs were designated key mRNAs. On the basis of these key mRNAs, we established a risk score formula:

$$\begin{aligned} \text{risk score} = & \mathrm{expFHOD}1*0.281350103316168 \\& +\mathrm{expORC}5* 0.565178944528756\\&+\mathrm{expTRIR}*(-0.46833132208101)\\&+\mathrm{expALKBH}1*(-0.460320304053552)\\&+\mathrm{expEPS}8L2*0.264637000551902\\&+\mathrm{expMBLAC}1*(-0.307676483709555)\\&+\mathrm{expMYADM}*0.148010675659352\\&+\mathrm{expHTRA}2*(-0.402279883752332)\\&+ \mathrm{expSRI}*(-0.627692885339051). \text{In the formula},\\& \text{"exp" refers to the expression levels of the mRNAs.}\end{aligned}$$

### Validation of the prognostic signature

**Fig. 3:**
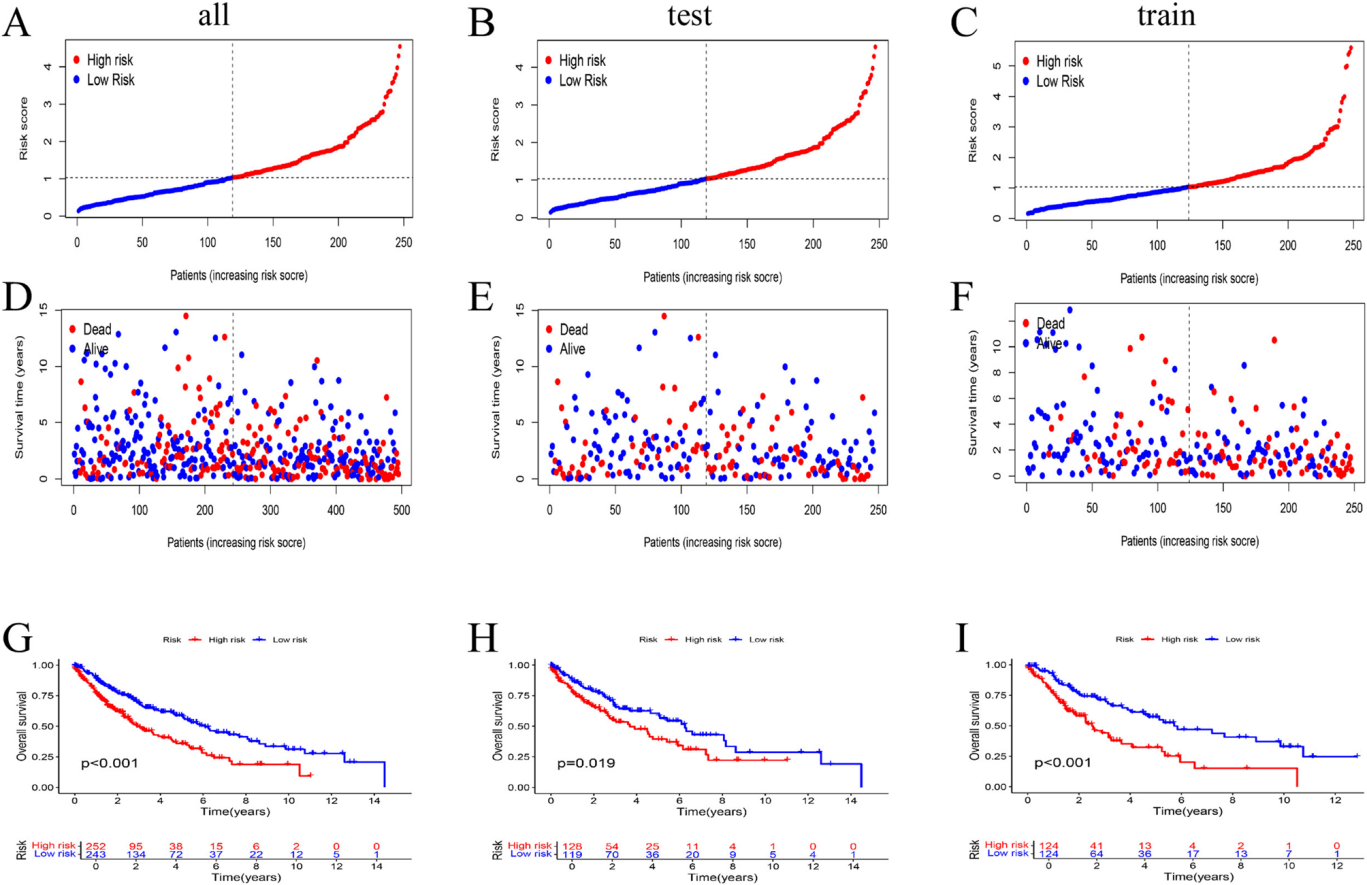
Validation of the prognostic signature. (A-C) Risk score distributions of the all, test and tain sets. (D-F) Survival status of the all, test and tain sets. (G-I) The all, test and train sets Kaplan–Meier curves

Figure 3A‒F compares the relationships between survival status and the risk score across different risk groups. In panels A-C, blue dots represent low-risk patients, whereas red dots indicate high-risk patients. In figure D-F, the dashed line divides patients into high- and low-risk groups, with low-risk patients on the left and high-risk patients on the right. The red dots represent deceased patients, while the blue dots represent survivors, with the y-axis showing survival time. We observed that, in all three groups, high-risk patients had a lower survival rate than low-risk patients did. K‒M curves revealed that the prognosis for the low-risk group (LRG) was better than that for the high-risk group (HRG) in all three groups (*P* < 0.05) (Fig. 3G-I).

### Independent prognostic value and predictive ability of the risk score model in LUSC

**Fig. 4:**
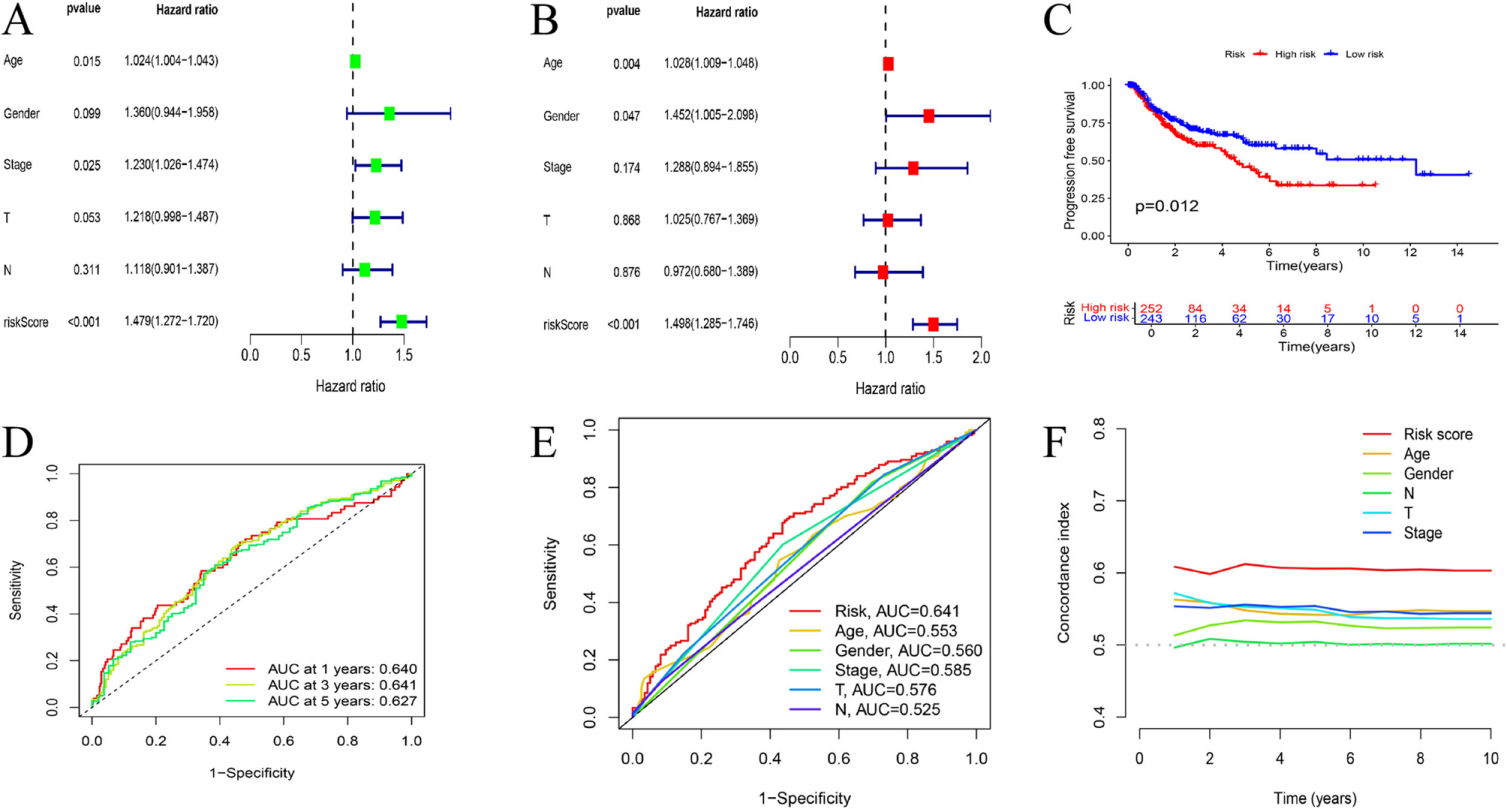
Independent Prognostic Value and Predictive Ability of the Risk Score Model in LUSC. (A-B) Results of univariate and multivariate Cox regression analyses of clinical characteristics. (C) PFS of all sets. (D) ROC curves for 1-, 3-, and 5-year survival in the ALL cohort. (E) ALL set ROC curves for the risk score, age, sex, stage, T stage and N stage. (F) C-index curves of the ALL cohort

We used univariate and multivariate Cox regression analyses to determine whether the risk score (RS) model could serve as an independent prognostic factor (Fig. 4A, B). We considered factors such as sex, age,, T stage, N stage, stage, and the risk score. The results indicated that the RS, age, and gender could all act as independent prognostic factors (*P* < 0.05). K‒M survival analysis based on the RS revealed that patients in the high-risk group (HRG) had significantly worse progression-free survival (PFS) than those in the low-risk group (LRG), suggesting that HRG patients have faster LUSC progression and a worse prognosis (Fig. 4C). We assessed the predictive accuracy of the model via ROC curves and the C-index. Time-dependent ROC curves revealed that the areas under the curves (AUCs) for 1-year, 3-year, and 5-year survival were 0.640, 0.641, and 0.627, respectively (Fig. 4D). Compared with other clinical factors (age, stage,, sex, T stage and N stage), the AUC values indicated that the RS has better predictive value (Fig. 4E). The C-index also suggested that the RS has superior predictive ability compared with other clinical characteristics (Fig. 4F).

### Validation of the broad applicability of the risk score model

**Fig. 5:**
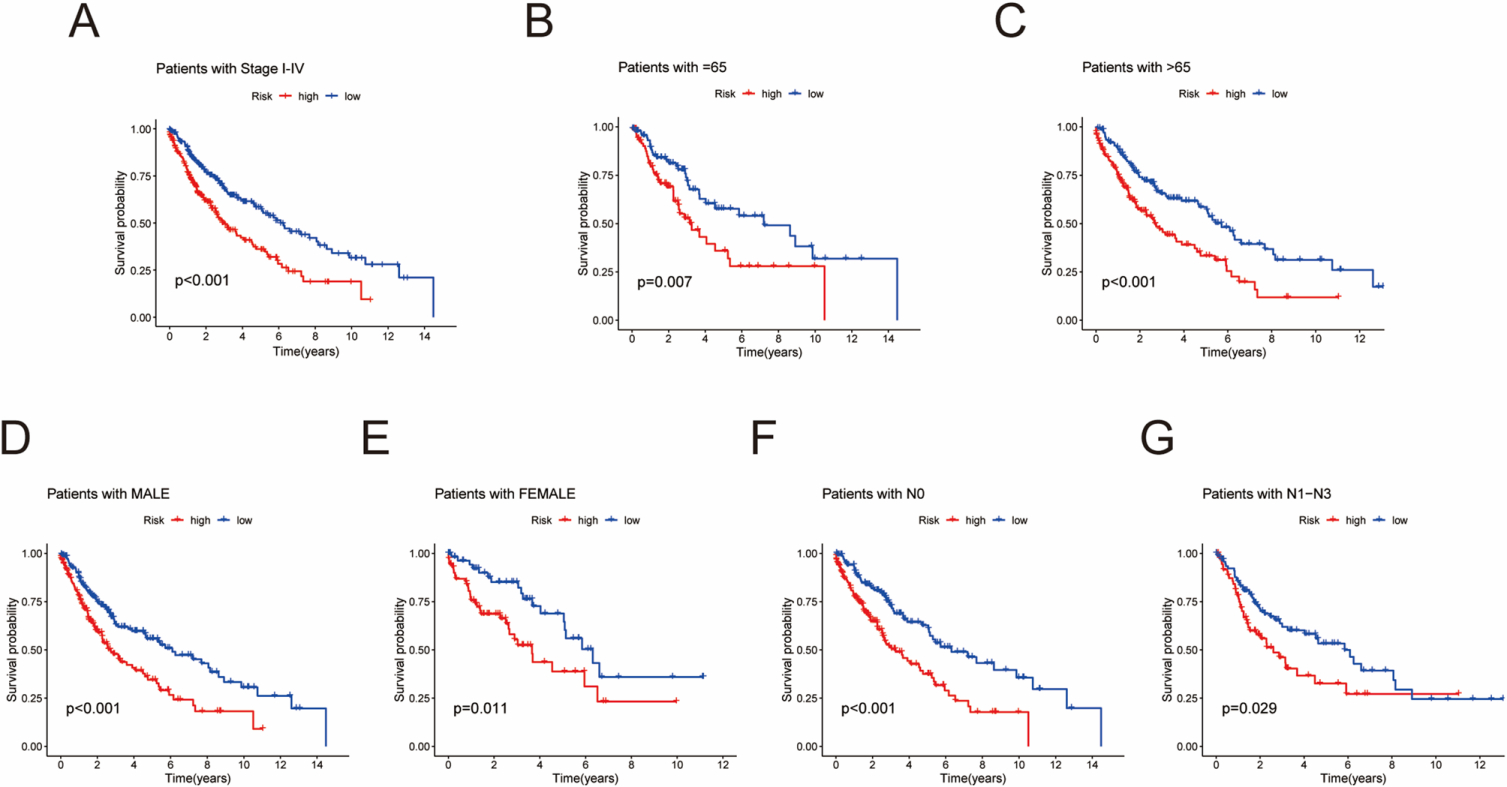
Validation of the Broad Applicability of the Risk Score Model. (A) Stage I-IV subgroups. (B) The subgroup of patients aged ≤ 65 years. (C) The subgroup with an age > 65 years. (D) Male subgroup. (E) Female subgroup. (F). The subgroups N0. (G). The subgroups N1-N3

We compared the survival rates of HRG and LRG across different clinical characteristics to assess the clinical applicability of the risk score (RS) model. The results revealed that for various clinical factors (stage, age, sex, and N stage), the survival status of HRG patients was worse than that of LRG patients (*P* < 0.05), indicating that the model has a broad range of applicability (Fig. 5A‒G).

### Functional enrichment analysis

To investigate the mechanism by which the RS model predicts patient prognosis, we conducted enrichment analysis to identify functional and pathway differences between HRG and LRG. GO enrichment analysis revealed that DEGs were primarily enriched in immune-related processes. KEGG analysis demonstrated that DEGs were mainly enriched in metabolism-related biological pathways and immune pathways (Supplementary Fig.S2).

**Fig. 6:**
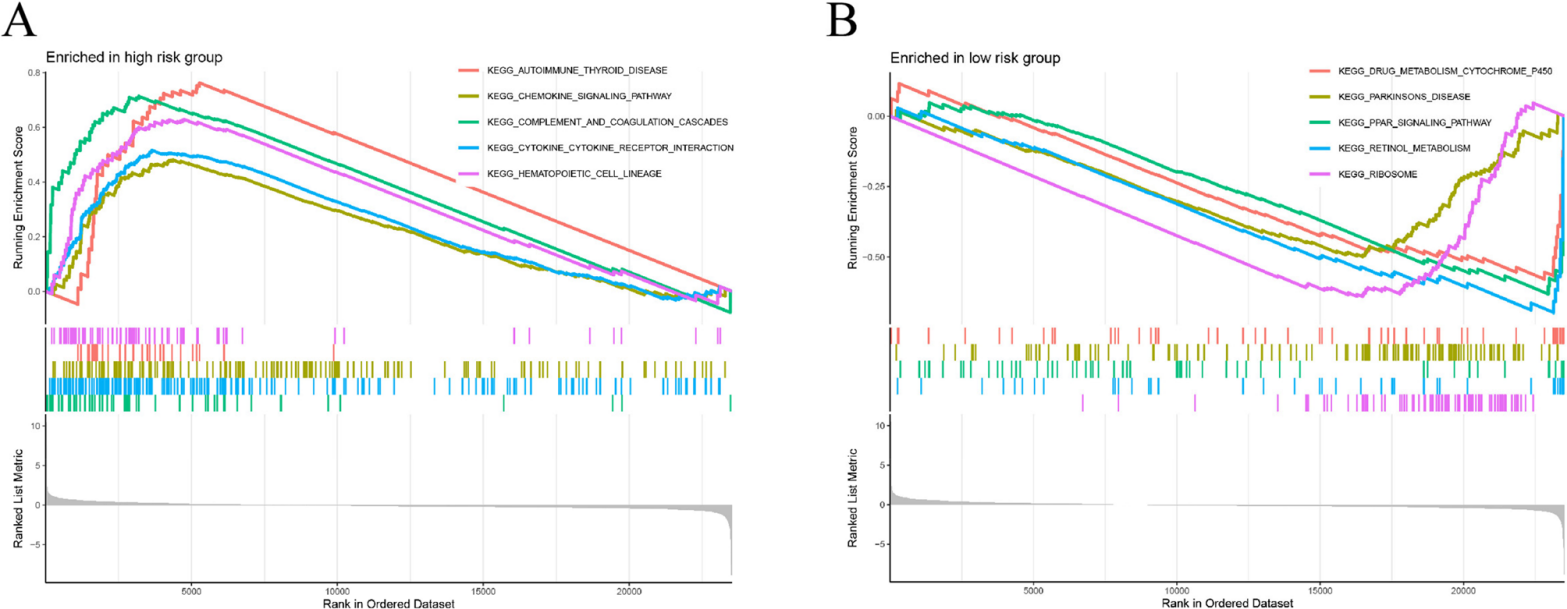
Functional enrichment analysis. (A-B) GSEA results for HRG and LRG

The GSEA results revealed that the high-risk set was enriched mainly in cytokine and chemokine signaling pathways (cytokine–cytokine–receptor interaction (KEGG), KEGG-chemokine signaling pathway) (Fig. 6A), whereas the low-risk set was enriched primarily in the PPAR signaling and cytochrome P450 metabolic pathways (Fig. 6B). Previous studies have indicated that PPAR signaling can inhibit tumor growth in non-small cell lung cancer, primarily by blocking the production of angiogenic ELR + CXC chemokines [18].

### Relationship between the RS and the tumor immune microenvironment (TIME)

**Fig. 7:**
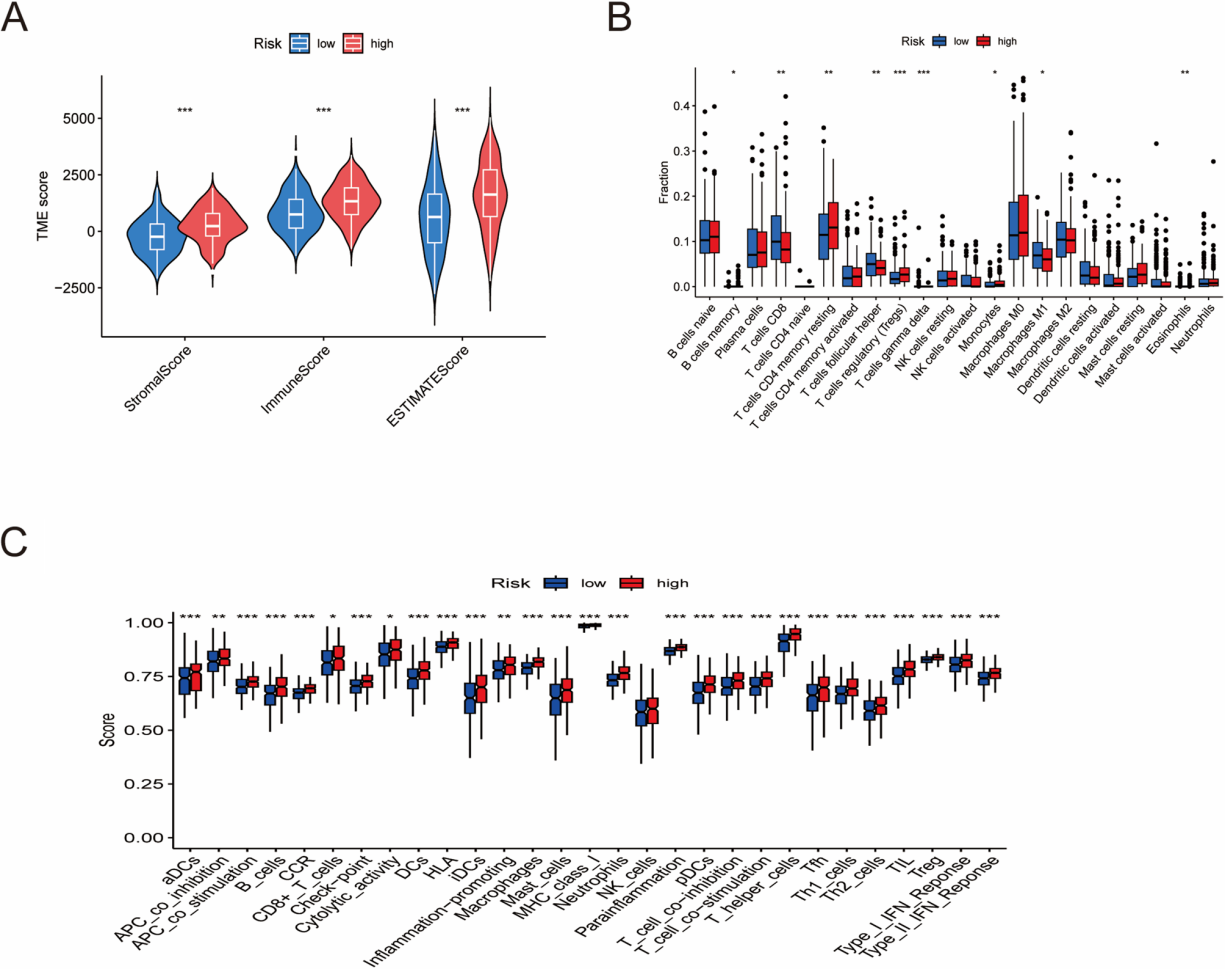
Relationship between the RS and the tumor immune microenvironment (TIME). (A) Results of ESTIMATE analysis for HRG and LRG. (B) Enrichment scores of 22 types of immune cells in the HRG and LRG. (C) Enrichment scores of 22 types of immune-related pathways were compared between the low- and high-risk groups

Compared with the LRG, the HRG has higher stromal and estimated scores, suggesting that the tumor microenvironment (TME) in the HRG is more complex (Fig. 7A). We used the CIBERSORT algorithm to assess the infiltration levels of 22 immune cell types in different risk groups. The results revealed that CD8 T cells, follicular helper T cells, and M1 macrophages were more highly enriched in the LRG, whereas resting CD4 memory T cells, regulatory T cells, and monocytes were more highly enriched in the HRG (Fig. 7B). This finding also indicates that there are differences in immune cell infiltration between the risk groups. Using the ssGSEA algorithm, we evaluated the immune function of the LRG and HRG, and the results showed that the HRG generally exhibited more active immune function than the LRG did (Fig. 7C).

### Correlation between the RS score and TMB

**Fig. 8:**
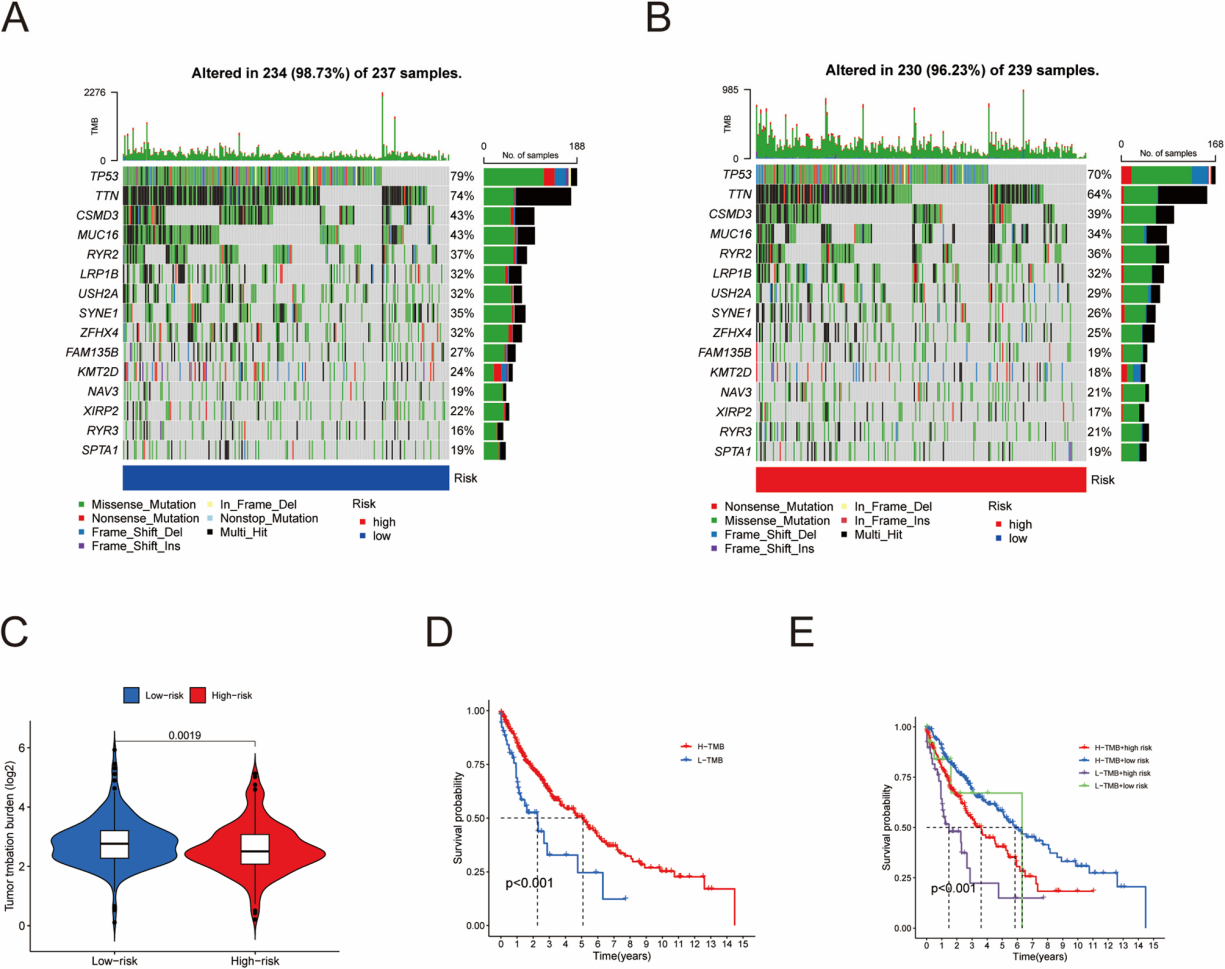
Correlation between the RS score and TMB. (A-B) Tumor mutation waterfall plots of the HRG and LRG. (C) Violin plot of the difference in TMB values between the HRG and LRG. (D) Survival rate differences between the high tumor mutation load group and the low tumor mutation load group. (E) Survival curves of LUSC patients with different RSs and TMBs

The TMB represents the number of DNA mutations per megabase (Mut/Mb) sequenced in a specific cancer [19]. It is a biomarker that can serve as a predictive factor for the response to immune checkpoint inhibitors (ICIs). Studies have shown that patients with high TMB tend to have better prognoses [20, 21]. Therefore, we analyzed the survival rate of LUSC patients on the basis of two variables, RS and TMB. There were no significant differences in the top 15 most mutated genes between HRG and LRG (Fig. 8A-B). Additionally, the TMB value of the LRG group was significantly greater than that of the HRG group (Fig. 8C). First, we divided the samples into two groups, H-TMB and L-TMB, on the basis of the median TMB of all samples to compare their prognosis. K‒M curves revealed that the survival rate of the H-TMB group was greater than that of the L-TMB group (Fig. 8D). We subsequently divided the samples into four groups, “H-TMB + H-risk,” “H-TMB + L-risk,” “L-TMB + H-risk,” and “L-TMB + L-risk,” to compare survival rates. The results indicated that the “L-TMB + H-risk” group had the worst prognosis, followed by the “H-TMB + H-risk” and “L-TMB + L-risk” groups, whereas the “H-TMB + L-risk” group had the best prognosis (Fig.8E). These findings further confirm the ability of the RS model to predict patient prognosis and suggest that our model can be applied in conjunction with the TMB to predict patient outcomes.

### Drug sensitivity analysis

**Fig. 9:**
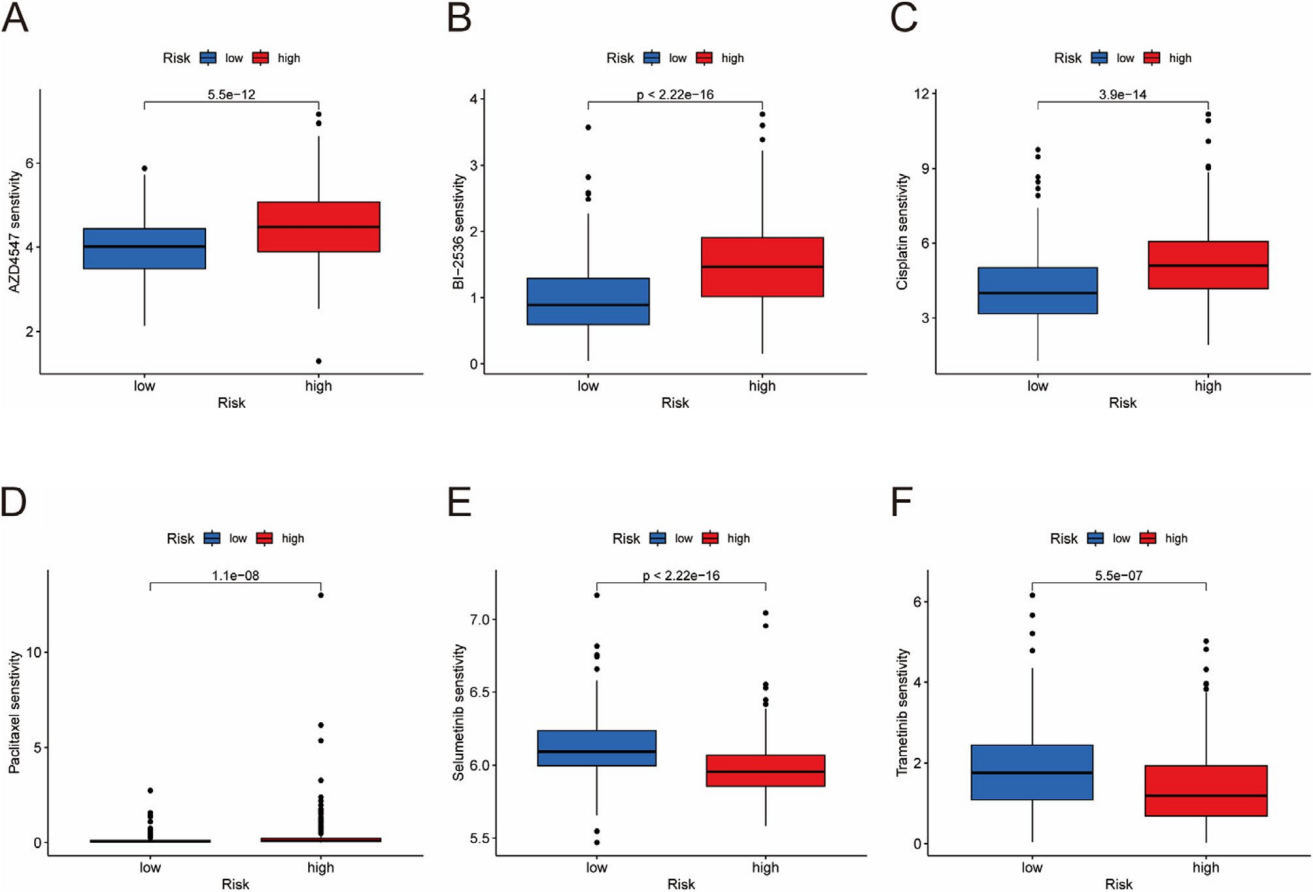
Drug sensitivity analysis. (A-F) Differences in the sensitivity of HRG and LRG to multiple drugs (selumetinib, paclitaxel, trametinib, cisplatin, AZD4547, and BI-2536)

We calculated the IC50 (half-maximal inhibitory concentration) values for each drug in the HRG and LRG using drug sensitivity data from the GDSC database, where a lower IC50 value indicates greater drug sensitivity. We compared the drug sensitivity differences for 70 chemotherapy drugs between the HRG and LRG, with 54 drugs being more sensitive to the LRG and 16 drugs being more sensitive to the HRG (Supplementary Table S3). We selected 6 commonly used drugs (selumetinib, paclitaxel, trametinib, cisplatin, AZD4547, and BI-2536) for visualization [22]. Among these, selumetinib and trametinib may have better therapeutic effects on HRG patients, whereas paclitaxel, cisplatin, AZD4547, and BI-2536 may be more effective for LRG patients (Fig. 9A-F).

### Experimental confirmation of the crucial function of ORC5 in lung squamous cell carcinoma

**Fig. 10:**
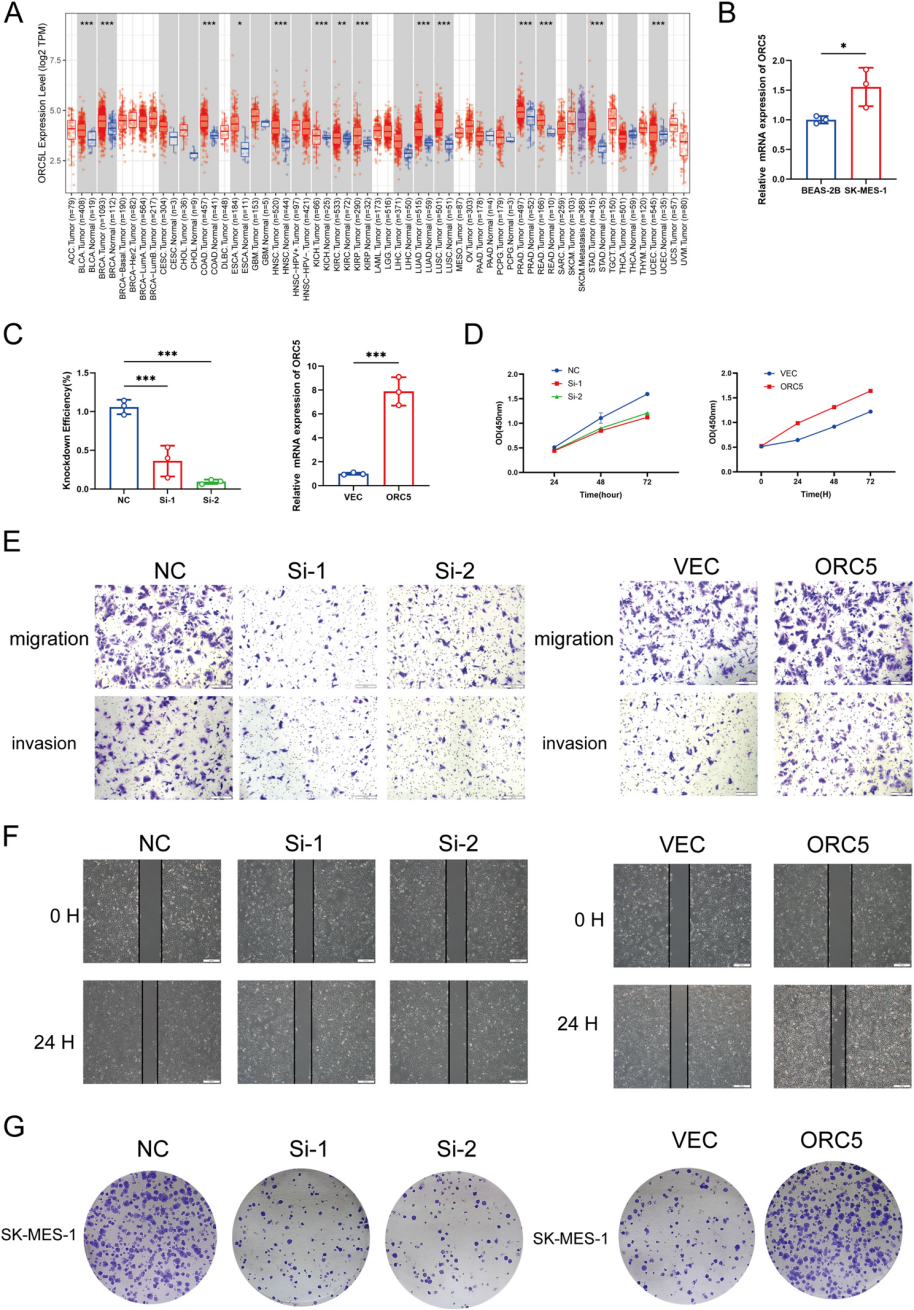
Experimental confirmation of the crucial function of ORC5 in Lung squamous cell carcinoma. (A) Differential expression analysis of ORC5 mRNA between tumor and adjacent normal tissues across multiple cancer types from TCGA database (TIMER2.0). (B) Q-PCR analysis of ORC5 expression in lung squamous cancer cells and normal lung epithelial cells. (C) Q-PCR confirmation of the knockdown and overexpression efficiency of ORC5. (D) CCK-8 results. (E) Wound-healing assay results. (F) Transwell assay results (scale bar: 100 μm). (G) Colony formation assay results. * *P* < 0.05, ** *P* < 0.01, *** *P* < 0.001

Using TIMER2.0, we retrieved the expression levels of ORC5 across various tissues from the TCGA database. Our analysis revealed a significant difference in ORC5 expression between lung cancer tissues and normal tissues. Furthermore, ORC5 exhibited differential expression in several other cancer types compared to their corresponding normal tissues, including bladder urothelial carcinoma, invasive breast carcinoma, and colon adenocarcinoma (Fig. 10A). On the basis of the above analysis, we measured the expression levels of ORC5 in BEAS-2B and SK-MES-1 cells. The results revealed that the expression level of ORC5 was significantly greater in SK-MES-1 cells than in normal cells (Fig. 10B). To further investigate the critical role of ORC5 in LUSC, we performed ORC5 knockdown and overexpression experiments in SK-MES-1 cells. The Q-PCR results confirmed the efficiency of ORC5 knockdown and overexpression (Fig. 10C). Through Cell Counting Kit-8 (CCK-8) assays, we found that reducing ORC5 inhibited cell proliferation, whereas overexpressing ORC5 promoted cell proliferation (Fig. 10D). Additionally, wound healing and transwell invasion assays demonstrated that ORC5 knockdown suppressed the invasive ability of the cells, whereas ORC5 overexpression had the opposite effect (Figs. 10E-F). Furthermore, colony formation assays revealed that ORC5 knockdown inhibited tumorigenic potential, whereas ORC5 overexpression enhanced colony formation (Fig. 10G). These experimental results indicate that ORC5 is a key molecule in tumor growth and metastasis and may contribute to poor prognosis in patients with lung squamous cell carcinoma.

## Discussion

Lung squamous cell carcinoma (LUSC), a major subtype of non-small cell lung cancer (NSCLC), remains a therapeutic challenge due to limited treatment options and poor prognosis [23]. In this study, we established a novel nine-gene prognostic signature based on disulfidptosis-related mRNAs, integrating multi-omics data and experimental validation to provide critical insights into risk stratification, immune microenvironment modulation, and therapeutic response prediction in LUSC. These findings advance our understanding of LUSC heterogeneity and lay a foundation for precision medicine strategies [24–26].

Disulfidptosis, a recently identified form of metabolic stress-induced regulated cell death (RCD), is mechanistically distinct from apoptosis or ferroptosis [27]. Under glucose deprivation, SLC7A11-overexpressing cancer cells experience NADPH deficiency due to impaired pentose phosphate pathway activity, coupled with SLC7A11-mediated cystine uptake. This leads to redox imbalance, accumulation of unreduced disulfides, and subsequent cytoskeletal collapse via abnormal crosslinking of actin proteins [24, 27]. Notably, tumors reliant on SLC7A11-mediated cystine metabolism exhibit heightened vulnerability to disulfidptosis in nutrient-deprived microenvironments, offering a novel therapeutic avenue [24, 27]. The discovery of disulfidptosis not only expands our understanding of cellular stress responses but also suggests new therapeutic opportunities for selectively targeting tumors with high SLC7A11 expression through metabolic interventions that disrupt redox homeostasis [28]. Our prognostic model, comprising FHOD1, ORC5, TRIR, ALKBH1, EPS8L2, MBLAC1, MYADM, HTRA2, and SRI, demonstrated robust predictive performance across cohorts (AUC:0.641), outperforming traditional clinical parameters (age, stage, sex, T stage and N stage). Multivariate Cox regression confirmed its independence as a prognostic factor, underscoring the clinical relevance of disulfidptosis-related genes in LUSC [17].

Functional enrichment analyses revealed distinct biological pathways associated with the high- and low-risk groups [29]. High-risk patients exhibited enriched cytokine-cytokine receptor interactions and chemokine signaling, aligning with the protumorigenic role of chronic inflammation [30] Conversely, the low-risk group exhibited activation of PPAR signaling and cytochrome P450-mediated metabolic pathways, which are known to suppress tumorigenesis by inhibiting angiogenesis and promoting detoxification [18]. These findings highlight the dual interplay of immune and metabolic reprogramming underlying LUSC heterogeneity. It provides an idea for immunotherapy, which is expected to improve the prognosis and management of patients. At the same time, our findings may contribute to the development of immunotherapy for LUSC. Tumor immune microenvironment (TIME) analysis further elucidated the mechanistic basis of the RS model. High-risk patients display elevated stromal and ESTIMATE scores, indicative of an immunosuppressive microenvironment characterized by resting CD4 + memory T cells and regulatory T cells [31]. In contrast, low-risk patients showed increased infiltration of cytotoxic CD8 + T cells and M1 macrophages, which are associated with enhanced antitumor immunity. These results align with the observed survival advantage in the low-risk group and underscore the interplay between disulfidptosis-related genes and immune evasion mechanisms [26].

The integration of TMB with the RS provides additional prognostic granularity [32]. While TMB alone correlated with improved survival, its combination with the RS stratified patients into four distinct prognostic subgroups. The “H-TMB + L-risk” group presented the most favorable outcomes, suggesting that the TMB and RS synergistically refine prognosis prediction. This finding holds clinical relevance, as TMB is an established biomarker for the immune checkpoint inhibitor (ICI) response [33]. The observed differences in TMB between risk groups may further guide immunotherapy strategies, although prospective validation is warranted.

Drug sensitivity analysis revealed differential responses to chemotherapy agents between risk groups [34]. High-risk patients show heightened sensitivity to MEK inhibitors (e.g., selumetinib and trametinib), potentially due to RAS/RAF pathway activation in aggressive tumors [35]. Conversely, low-risk patients benefit more from conventional agents such as cisplatin and paclitaxel, which is consistent with their association with immune-active microenvironments. These findings emphasize the utility of the RS model in guiding personalized therapeutic regimens [36].

Compared to normal tissue, ORC5 exhibits significant differential expression in multiple cancer types. Particularly in lung cancer tissue, ORC5 expression is substantially higher than in normal tissue, indicating its association with LUSC development. Experimental validation of ORC5 (a critical component of the origin recognition complex) has revealed its oncogenic role in LUSC [32, 33]. As a core subunit of the origin recognition complex (ORC), ORC5 regulates DNA replication initiation by recruiting CDC6/MCM complexes and maintains chromatin stability via spatial regulation of HP1α [15, 37, 38]. Overexpression of ORC5 correlated with poor prognosis (HR = 2.15, *P* = 0.003), and its knockdown suppressed SK-MES-1 cell proliferation (54%) and invasion (67%), consistent with its role in hepatocellular carcinoma progression [39]. Under replication stress, ORC5 interacts with Rrm3 to modulate DNA synthesis, suggesting dual roles in genome stability [40, 41]. While the mechanistic link between ORC5 and disulfidptosis requires further investigation, its functional significance reinforces the biological plausibility of our model.

### Limitations and Future Directions

Despite these advances, certain limitations warrant consideration. First, the retrospective nature of TCGA data necessitates validation in prospective cohorts. Second, the precise mechanisms linking disulfidptosis-related genes to immune modulation (e.g., T-cell exhaustion, macrophage polarization) remain unclear. Third, clinical translation of the risk model, particularly in predicting ICI responses, requires further investigation. Finally, while our prognostic model demonstrates robust predictive performance across multiple datasets, we acknowledge that functional validation of representative genes—particularly those with opposing expression patterns—was not performed in this study. To address this limitation and further strengthen the clinical relevance of our findings, future studies should prioritize experimental validation of these candidate genes. For example, single-cell sequencing could be employed to dissect cell-type-specific expression patterns, while organoid models might help screen potential disulfidptosis-targeting compounds. Such approaches would not only validate the mechanistic roles of the signature genes but also facilitate translational applications.

## Conclusions

In this study, we developed a prognostic model for LUSC based on disulfidptosis-related mRNAs and investigated the relationships between this model and the immune microenvironment, TMB, and drug sensitivity. The model enables the assessment of prognosis and treatment efficacy in LUSC patients and offers new perspectives for the treatment of LUSC.

## Supplementary Information

Supplementary material 1: Table S1

Supplementary material 2: Table S2

Supplementary material 3: Table S3

Supplementary material 4: Figure S1

Supplementary material 5: Figure S2

## Data Availability

The TCGA database is located at https://portal.gdc.cancer.gov, accessed on 3 December 2022, accession number: TCGA-LUSC; the GSEA database is located at https://www.gsea-msigdb.org.

## Abbreviations

LUAD: Lung adenocarcinoma
LUSC: Lung squamous cell carcinoma
RCD: Regulated cell death
ACD: Accidental cell death
DRGs: Disulfidptosis-related genes
PCa: Pancreatic cancer
CCRCC: Clear Cell Renal Cell Carcinoma
LASSO: Least Absolute Shrinkage and Selection Operator
LRG: Low-risk groupand
HRG: High-risk group
PFS: Progression-free survival
FDR: false discovery rate
GO: Gene Ontology
KEGG: Kyoto Encyclopedia of Genes and Genomes
GSEA: Gene Set Enrichment Analysis
TMB: Tumor mutation burden
siRNA: Small interfering RNA
CCK-8: Cell Counting Kit-8
PBS: phosphate-buffered saline
