# Correction: A novel disulfidptosis-related mRNA signature predicts prognosis and therapeutic response in lung squamous cell carcinoma

**DOI:** 10.1186/s12890-026-04133-1

**Published:** 2026-02-19

**Authors:** Wei Bai, Ning Jiang, Yuhan Deng, Xiaofeng Tang, Feifei Zhang, Shaorui Niu, Yuyang Yao, Yuhao Zhou, Kangming Chen, Liping Li, Jun Yang, Xiao-Bin Lv

**Affiliations:** 1 School of Public Health, Jiangxi Medical College, Nanchang University, Nanchang, 330006 China; 2 Jiangxi Provincial Key Laboratory of Disease Prevention and Public Health, Nanchang University, Nanchang, 330006 China; 3 Jiangxi Key Laboratory of Oncology, The Third Affiliated Hospital, Jiangxi Medical College, The Central Lab of The First Hospital of Nanchang, Nanchang University, North 128 Xiangshan Road, Nanchang, 330008 China; 4 Institute (College) of Integrative Medicine, Dalian Medical University, Dalian, 116044 China; 5 College of Integrated Chinese and Western Medicine, Hunan University of Chinese Medicine, Changsha, 410208 China

**Correction: BMC Pulm Med 25**,** 462 (2025)**

10.1186/s12890-025-03920-6

Following publication of the original article [1], an error was identified in the Conclusions section. The updated conclusion is given below and the changes have been highlighted in **bold typeface**.

**1. In Fig. 4**,** C is missing from the legend.**

Figure 4 Independent Prognostic Value and Predictive Ability of the Risk Score Model in LUSC. (A-B) Results of univariate and multivariate Cox regression analyses of clinical characteristics. **(C) PFS of all sets**. (D) ROC curves for 1-, 3-, and 5-year survival in the ALL cohort. (E) ALL set ROC curves for the risk score, age, sex, stage, T stage and N stage. (F) C-index curves of the ALL cohort.

**2. Figure 10 has many legend-image mismatches.**

- **Legend-image mismatch**:

Figure 10 Experimental confirmation of the crucial function of ORC5 in Lung squamous cell carcinoma. (A) Differential expression analysis of ORC5 mRNA between tumor and adjacent normal tissues across multiple cancer types from TCGA database (TIMER2.0). (B) Q-PCR analysis of ORC5 expression in lung squamous cancer cells and normal lung epithelial cells. **(C) Q-PCR confirmation of the knockdown and overexpression efficiency of ORC5**. (D) CCK-8 results. (E) Wound-healing assay results. (F) Transwell assay results (scale bar: 100 μm). (G) Colony formation assay results. * *P* < 0.05, ** *P* < 0.01, *** *P* < 0.001.

b) **Incorrect reference in Results section, “Experimental confirmation of the crucial function of ORC5 in lung squamous cell carcinoma” sub section:**

Furthermore, colony formation assays revealed that ORC5 knockdown inhibited tumorigenic potential, whereas ORC5 overexpression enhanced colony formation **(Fig. 10G)**. These experimental results indicate that ORC5 is a key molecule in tumor growth and metastasis and may contribute to poor prognosis in patients with lung squamous cell carcinoma.

**3. Incorrect references in the Results section**,** “Functional enrichment analysis” subsection**:

The GSEA results revealed that the high-risk set was enriched mainly in cytokine and chemokine signaling pathways (cytokine–cytokine–receptor interaction (KEGG), KEGG-chemokine signaling pathway) **(Fig. 6A)**, whereas the low-risk set was enriched primarily in the PPAR signaling and cytochrome P450 metabolic pathways **(Fig. 6B)**. Previous studies have indicated that PPAR signaling can inhibit tumor growth in non-small cell lung cancer, primarily by blocking the production of angiogenic ELR + CXC chemokines [18].

**4. Typo in Fig. 3 Legend**:

Figure 3 Validation of the prognostic signature. (A-C) Risk score distributions of the all, test and **train** sets. (D-F) Survival status of the all, test and train sets. (G-I) The all, test and **train** sets Kaplan–Meier curves.

**5. Missing reference to Fig. 8E in the Results section**,** “Correlation between the RS score and TMB” subsection**:

We subsequently divided the samples into four groups, “H-TMB + H-risk”, “H-TMB + L-risk”, “L-TMB + H-risk”, and “L-TMB + L-risk”, to compare survival rates. The results indicated that the “L-TMB + H-risk” group had the worst prognosis, followed by the “H-TMB + H-risk” and “L-TMB + L-risk” groups, whereas the “H-TMB + L-risk” group had the best prognosis **(Fig. 8E)**. These findings further confirm the ability of the RS model to predict patient prognosis and suggest that our model can be applied in conjunction with the TMB to predict patient outcomes.

**6. Correction of an incorrect URL in the Materials and Methods section**,** “Data acquisition” subsection**:

We obtained patient data from the TCGA database **(**http://portal.gdc.cancer.gov.**)**, which included a total of 553 samples, comprising 51 normal samples and 502 tumor samples. Additionally, we collected clinical data from 504 samples, which were used for subsequent prognostic analysis. Furthermore, we gathered mutation data related to lung squamous cell carcinoma (LUSC) from the TCGA database to facilitate comprehensive molecular and clinical investigations.”

**7. Removal of an irrelevant Keyword**:

Incorrect: Lung squamous cell carcinoma, Disulfidptosis, Prognostic signature, Risk stratification, Tumor microenvironment **AUV**.

Correct: Lung squamous cell carcinoma, Disulfidptosis, Prognostic signature, Risk stratification, Tumor microenvironment.

**8. Renaming of Supplementary Materials**:

Supplementary Table S1 in the main text of the manuscript should be replaced with Supplementary Material 2.

Supplementary Table S3 in the main text of the manuscript should be replaced with Supplementary Material 3.

Supplementary Fig. S2 in the main text of the manuscript should be replaced with Supplementary Material 4.

Supplementary Fig. S1 in the main text of the manuscript should be replaced with Supplementary Material 5.

The original article has been corrected.
