# Supplementary figures and images for: A novel disulfidptosis-related mRNA signature predicts prognosis and therapeutic response in lung squamous cell carcinoma
Source: BMC Pulm Med. 2025 Oct 8;25:462. doi: 10.1186/s12890-025-03920-6 (PMC12505621; doi:10.1186/s12890-025-03920-6)

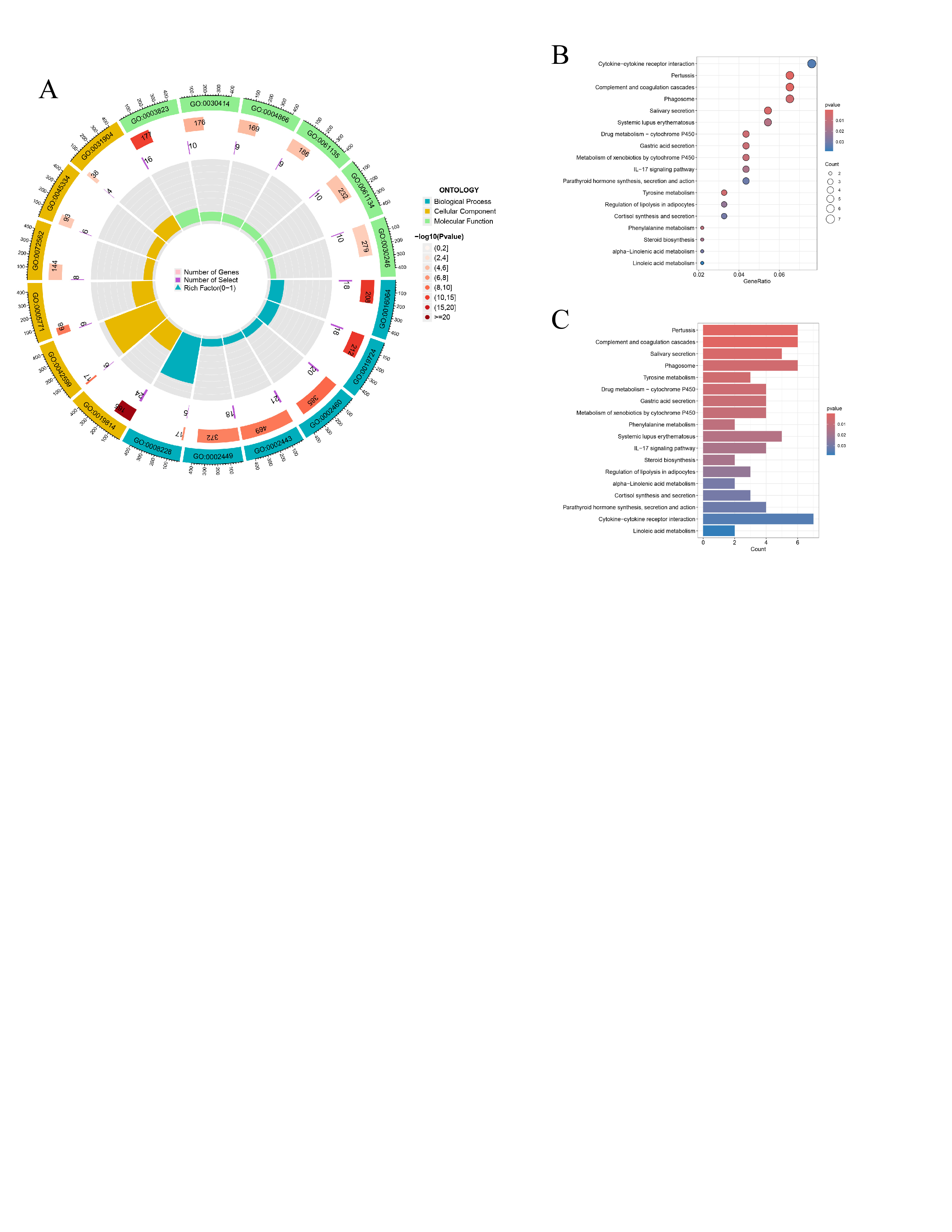

Supplement: Supplementary file 4 — Supplementary material 4: Figure S1 [file 12890_2025_3920_MOESM4_ESM.tif]
